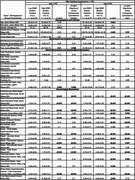# White Matter Hyperintensities burden is associated with higher plasma NfL and poorer global cognition in MCI: Findings from the Southeast Asian BIOCIS study

**DOI:** 10.1002/alz70856_101548

**Published:** 2025-12-25

**Authors:** Gurveen Kaur Sandhu, Jacklyn Leonardo, Fatin Zahra Zailan, Xin Ying Sim, Ashwati Vipin, Bocheng Qiu, Rasyiqah Binte Shaik Mohamed Salim, Pricilia Tanoto, Kiirtaara Aravindhan, Faith Phemie Hui En Lee, Smriti Ghildiyal, Shan Yao Liew, Gursimar Bhalla, Yi Jin Leow, Nagaendran Kandiah

**Affiliations:** ^1^ Dementia Research Centre (Singapore), Lee Kong Chian School of Medicine, Nanyang Technological University, Singapore, Singapore; ^2^ Lee Kong Chian School of Medicine, Nanyang Technological University, Singapore, Singapore; ^3^ Dementia Research Centre (Singapore), Lee Kong Chian School of Medicine, Nanyang Technological University, Singapore 308232, Singapore, Singapore; ^4^ Dementia Research Centre (Singapore), Lee Kong Chian School of Medicine, Nanyang Technological University, Singapore 308232, Singapore, Singapore, Singapore; ^5^ nil, nil, nil, Nicaragua; ^6^ Neuroscience and Mental Health Programme, Lee Kong Chian School of Medicine, Nanyang Technological University, Singapore, Singapore; ^7^ National Healthcare Group, Singapore, Singapore

## Abstract

**Background:**

NfL, a critical neuronal cytoskeleton structural protein has been demonstrated to be increased with the presence of White Matter Hyperintensities (WMH). Nevertheless, the effect of NfL expression in the context of WMH burden and cognition in younger versus older cognitively impaired adults, has yet to be completely elucidated. We therefore investigate this in a Southeast Asian mild cognitive impairment (MCI) community cohort having high burden of cerebrovascular disease.

**Method:**

A cross‐sectional study was conducted in 310 Southeast Asian community participants recruited into the Biomarker and Cognition Study and classified as MCI. The cohort was stratified by WMH burden, a surrogate measure for cerebrovascular disease, as quantified using the modified Fazekas scale (Fazekas et al., 1987; Vipin et al., 2021). Neuropsychological Assessments of global cognition tailored for multilingual populations, the Visual Cognitive Assessment Test (VCAT) was measured (Kandiah et al., 2016). Plasma NfL was quantified using the Single Molecule Array (Simoa) platform (Wilson et al., 2016). All means and associated proportions were calculated for all variables with comparative analysis conducted using independent samples T‐Tests/Chi‐square and controlled for significant confounding effects where applicable.

**Result:**

Preliminary findings indicate that high WMH burden in younger adults with MCI was significantly associated with higher NfL concentration, poorer global cognition score and larger perivascular spaces (Table 1). On the other hand, high WMH burden in older adults with MCI, was significantly associated with higher markers of cerebrovascular disease ‐ namely, systolic blood pressure, increased perivascular spaces, higher count of lacunar infarcts and medial temporal atrophy, together with decreasing gray matter (Table 1).

**Conclusion:**

NfL, a neurodegenerative measure is increased in a younger Southeast Asian community MCI population with high WMH burden. A plateau of this increase is observed in the older MCI population, which could be a result of threshold effects together with reduced neurobiological capacity. This makes NfL a promising biomarker for the early detection and prognostication of white matter disease and resultant cognitive impairment among Southeast Asians, allowing for the application of beneficial and timely interventions. Further mechanistic validation in a larger more pathologically stratified cohort, followed by longitudinal follow‐up is underway.